# Handgrip Strength Cut-Off Values for the Undernutrition Risk Screening among Elderly Men and Women in Bosnia and Herzegovina

**DOI:** 10.1155/2019/5726073

**Published:** 2019-11-03

**Authors:** Maja Račić, Jelena Pavlović, Nedeljka Ivković

**Affiliations:** ^1^Thomas J. Stephens & Associates Research Center, 3635 W Altadena Ave, Phoenix 85029, USA; ^2^Department of Nursing, Faculty of Medicine in Foca, University of East Sarajevo, Studentska 5, 7330 Foca, Bosnia and Herzegovina; ^3^Department of Oral Rehabilitation, Faculty of Medicine in Foca, University of East Sarajevo, Studentska 5, 7330 Foca, Bosnia and Herzegovina

## Abstract

**Objectives:**

To determine the optimal cut-off points of handgrip strength (HGS) to identify the undernutrition risk among individuals older than 65 years of age in Bosnia and Herzegovina.

**Design:**

Cross-sectional study.

**Setting:**

Towns of Sarajevo, Foca, Rogatica, and Pale in Bosnia and Herzegovina.

**Participants:**

300 community-dwelling older adults and 146 nursing home residents. Comprehensive Geriatric multidimensional assessment (CGA) was carried out to evaluate general health, functional, and cognitive capabilities. Nutritional status and undernutrition risk were assessed by Mini Nutritional Assessment (MNA) and Seniors in the Community: risk evaluation for eating and nutrition, version II (SCREEN II). HGS was measured with a Smedley dynamometer.

**Results:**

According to the classification of nutritional status by MNA, 42% of community-dwelling men and 39% of community-dwelling women were at undernutrition risk. The undernutrition risk was significantly higher among nursing home residing men (89%) and women (78%) (*p* < 0.001). When nutritional status was assessed by SCREEN II, 100% on nursing home residents, 86% of community-dwelling men and 80% of women were identified as having a high risk for undernutrition. Per MNA, HGS cut-off thresholds were 23.50 kgF (65–74 years) and 19.50 kgF (≥75 years) for men; 15.50 kgF (65–74 years) and 13.50 kgF (≥75 years) for women. Per SCREEN II, cut-points were 28.50 kgF (65–74 years) and 24.50 kgF (≥75 years) for men; 24.50 kgF (65–74 years), 19.50 kgF (≥75 years for women).

**Conclusion:**

HGS can be a useful instrument to identify undernutrition risk among the elderly patients. This study provides threshold for men and women older than 65 years of age in Bosnia and Herzegovina.

## 1. Introduction

Undernutrition among the elderly population is a prevalent public health problem that may ultimately lead to the progression of underlying chronic diseases and the development of acute conditions (dehydration, infection, and delirium) [1]. Undernourished older individuals have a lower quality of life, increased risk for frequent hospital admissions, higher mortality rates, and more significant health expenditures compared to their well-nourished peers [2]. Therefore, routine assessment of undernutrition risk is highly recommended in medical practice; once a year, for community-dwelling older people and at intervals, or 1–3 months for elderly inpatients [3–5].

According to Bedogni, an ideal screening instrument should include the analysis of risk factors, energy/nutrients balance, and anthropometric, functional, and biochemical indicators of undernutrition risk [6]. Although many screening instruments have been validated and implemented in hospitals or nursing homes, a majority of them either failed to encompass all recommended indicators or were too robust for application in a family practice setting (which generally provides primary health care for the community-dwelling elderly population throughout Europe) [2, 5]. A study on the attitudes of general practitioners toward barriers and opportunities for undernutrition risk assessment in their practices identified a lack of time, staff, resources, and knowledge as the main obstacles to implementing the process of screening in general practice. The administration of a short nutrition screening instrument during the medical encounter was identified as the facilitator of nutritional status screening uptake [7].

Although anthropometric parameters, such as body mass index (BMI) and circumferences, have been used widely as standard indicators of nutritional status, they often mask sarcopenia or other weight changes needed to evaluate undernutrition risk [8]. Recent studies regarded HGS as an independent predictor and the one of the earliest markers of undernutrition [9–21]. According to the meta-analysis of normative data, HGS values of 16 kgF for women and 27 kgF for men could be accepted as cut-off point for undernutrition risk across Europe, Canada, United States, Australia, and Japan [14]. In line with research of Charlton et al. conducted in South Africa [22], a Chinese study suggested 24.9 kgF for men and 15.2 kgF for women as the optimal HGS value for elderly people [12]. As skeletal muscle mass values vary between different populations and ethnicities, general references for HGS cannot be used, so it has been recommended to collect national HGS data and calculate its cut-off points [23]. To our knowledge, HGS cut-off points for the undernutrition risk have not been described so far for the Bosnian population.

The aim of this study was to determine the optimal cut-off values of HGS to identify the undernutrition risk among individuals older than 65 years of age in Bosnia and Herzegovina.

## 2. Material and Methods

### 2.1. Study Sample

The cross-sectional study was carried out in four municipalities of Bosnia and Herzegovina (East Sarajevo, Pale, Rogatica, Foca) between April and September 2018. Study participants were individuals older than 65 years of age, living in the community (community-dwelling) or nursing homes. Sample size calculation was conducted to assess the minimum number of prospective subjects. The calculation was based on the previous study, which showed that the prevalence of undernutrition in community-dwelling elderly is 15% and in the nursing home residents is 30% [4]. With a population size of 3900 elderly people, and an error of 5% and CI 95%, the sample consisted of 446 participants. The total population sampling technique was applied to select participants among nursing home residents. Community-dwelling participants were selected from the registry of family medicine patients. A computer program was employed to generate a list of random numbers and each patient was assigned a number. Inclusion criteria for both community-dwellers and nursing home residents were an orientation in time, space, and person. Critically ill persons; disoriented and uncooperative persons; and those with malformations, aphasia, dysphasia, severe mental illnesses, dementia, cancers, and chronic renal insufficiency were excluded from the study and then substituted with new participants to achieve the calculated sample size. Selected participants were contacted through their family physicians or nurses.

### 2.2. Data Collection

All clinical measurements were performed by the principal investigator, with the same instruments. To test reliability, the pilot study was carried out on the separate sample of 25 elderly individuals (not included in final analyses). Reproducibility of tests was analyzed by the interclass correlation coefficient (ICC). The rater performed all clinical measurements on 5 patients per day during five consecutive days. The measurements were repeated seven days later, by the same rater and on the same individuals. ICC estimates were between 0.798 and 0.886, so the level of intrarater reliability could be regarded as good. Research assistants and trained nursing students administered questionnaires. To maintain anonymity and confidentiality, no identifying information about participants was collected. The principal investigator explained the aims of the study and asked each participant to sign an informed consent form. This study was conducted according to the guidelines laid down in the Declaration of Helsinki, and all procedures involving research study participants were approved by the Ethics Committee of Faculty of Medicine Foca (No: 01-2-1). Written informed consent was obtained from all subjects/patients.

### 2.3. Instruments

#### 2.3.1. Comprehensive Geriatric Assessment

To meet the expert recommendations, clinical assessment of undernutrition risk included the evaluation of dietary, anthropometric, functional, and biochemical indicators [6, 23]. The sociodemographic questionnaire was designed for the study included information on gender, age, marital status, place of living, education, medications. Comprehensive Geriatric multidimensional assessment (CGA) was carried out to evaluate general health, functional, and cognitive capabilities. Ability to perform basic and instrumental activities of daily living (ADL) was assessed using the Katz Index [24] and Lawton Scale [25]. The researchers used the Bosnian versions of questionnaires. Katz index included bathing, toileting, dressing, transferring, continence, and feeding independence. If no assistance was needed, individuals were considered independent (1 point). The score ranged from 0–6, with lower scores indicating greater dependence. The Lawton Scale evaluated the ability of participants to live independently in the community (8 items) and was distributed to community-dwelling participants only. Items were rated on the scale from 0 (low functioning) to 8 (high functioning). Cronbach's alpha coefficients for the Katz Index and Lawton Scale in Bosnian language were considered good (*α* = 0.874) and excellent (*α* = 0.952).

To assess memory, calculation, and orientation, the Six-Item Cognitive Impairment test (6-CIT) was used. Scoring was inverse and included the following categories: <10 (normal cognitive functioning); 10–19 (mild cognitive impairment); ≥20 (significant cognitive impairment) [26]. According to Cronbach's coefficient value of 0.827, the reliability of 6-CIT was good.

Timed Up and Go Test was performed to assess mobility. A standard chair with support for the spine was used. The participants were required to rise from the chair, walk at a normal pace 3 m to a line on the floor, turn, walk back to the chair, and sit down, without any encouragement from the investigator. In one-minute intervals, three tests were completed and measured in seconds (stopwatch). The best time recorded was considered as the performance score for mobility. The thresholds were categorized as follows: <10 s (normal); 11–20 s (good mobility); and >21 s (impaired mobility) [27].

The functional reach test was carried out with the participants standing with their dominant arm next to the wall, placed at 90 degrees of shoulder flexion, with elbows and hands extended. Fingers were closed into a fist. The starting position was recorded at the tip of the middle finger on the measuring tape. The participants were instructed to reach as far as they could without taking a step or lowering their arm. The test was repeated three times with a pause of 30 sec in between. The difference between the starting and reached point was calculated in centimeters, and the mean value was recorded. The threshold for impaired mobility was <15 [28].

#### 2.3.2. Nutritional Status Assessment

Likelihood of undernutrition was assessed by Mini Nutritional Assessment (MNA). MNA included 18 items grouped into screening (food intake, weight loss, mobility, suffered psychological stress, and neuropsychological problems) and assessment scales (independence, medications, pressure sore's presence, and protein intake). Total indicator scores were categorized as undernutrition (<17); a risk of undernutrition (17–23.9); and normal nutritional status (≥24) [29]. Internal consistency was calculated, and Cronbach's *α* coefficient of 0.726 indicated adequate reliability of MNA.

Likelihood of undernutrition risk was evaluated using seniors in the community: risk evaluation for eating and nutrition, version II [SCREEN II). SCREEN II consisted of 14 items (each with five possible score from 0–4), providing information on weight change (1 item), meal frequency (2 items), appetite (1 item), food intake (4 items), and risk factors (6 items), such as difficulties with chewing, swallowing, meal preparation or groceries shopping, eating alone, and meal replacement. The total number of points ranged from 0 to 64, with the score lower than 50 indicating high, 50–54 moderate, >54 low nutritional risk [30]. Internal consistency of SCREEN II was fair (*α* = 0.610). The SCREEN II questionnaire is the copyright of Dr. Heather Keller.

#### 2.3.3. Anthropometric Measurement

Duplicate measurement of weight (to the nearest 0.1 kg) and height (to the nearest 0.5 cm) were taken by a standard weighbridge and stadiometer, respectively. To obtain a body mass index (BMI) value (kg/m^2^), weight was divided by the square height. With a standard nonelastic tape measure, calf circumference (CC) was measured at the level of the maximum gastrocnemius muscle's bulk when in a standing position on both legs. Compression of subcutaneous tissue was avoided. Midupper arm circumference measurements were taken on both arms while relaxed at the side with palms facing inwards (midpoint between the olecranon and acromial process), and following the contour of the tissue without compressing it. Measurement of waist circumference was performed in the horizontal plane midway between the lowest rib and the iliac crest, at the end of expiration. To assess reproducibility, anthropometric measurements were repeated three times, and the average values were recorded in centimeters [23].

Skinfold thickness measures were taken in standing position, on the right arm. The investigator picked up skinfold (including the subcutaneous layer) between the thumb and middle finger and pulled it away from the muscle tissue. The edges of a GIMA code 27320 Caliper were placed 1 cm below the investigator's fingers. The caliper (readings were executed 3 seconds after full pressure was applied to the nearest 1 mm). The procedure included four sites: triceps (vertical fold at the posterior midline of the upper arm halfway between acromion and olecranon process), biceps (vertical fold at the anterior midline of the upper arm), subscapular (diagonal fold at interior angle of the scapula), and suprailiac (diagonal fold, 1 cm above the anterior superior iliac crest). All measurements were done in triplicate, and the mean value was recorded as a score of skinfold thickness. The scores were converted into a total fat percentage (% BF) [23].

#### 2.3.4. Biochemical Nutritional Markers

Blood markers included albumin, hemoglobin, total cholesterol, and ferritin. All parameters were measured according to the protocols of Laboratory for hematological, immunological, and biochemical analyses.

#### 2.3.5. HGS Measurement

HGS was measured using calibrated Smedley dynamometer. The participants were seated with elbows by their sides flexed at a 90° angle, supported in a neutral wrist position. The investigator asked participants to squeeze the dynamometer as hard as they could for 3 sec. Measurements were carried out three times at intervals of 1 minute on the right and left arm. The maximal value was recorded in kilograms. HGS of the dominant hand was used for analysis. For Smedley dynamometer, the force range was 1–100 kilograms, and the resolution was 0.5 kgF [31]. As body height contributes independently to muscle strength, HGS values were stratified by age and height.

#### 2.3.6. Statistical Analysis

Statistical analyses were performed using the Statistical Package for Social Science version 25 (SPSS, IBM, Inc. Chicago, IL, US). Categorical variables were presented as numbers and percentages; numerical variables, as means and SD, medians and percentiles. Percentiles were stratified by age, gender, and body height. To test the difference between the variables, Chi-square, or Kruskal–Wallis test for nonparametric and *T*-test or Analysis of variance (ANOVA) for numerical variables were applied. Pearson's correlation coefficients were used to measure the extent to which HGS is related to other variables. The ROC curves were generated to determine sensitivity and specificity of optimal cut-off values for handgrips of the dominant arm in relation to MNA (<17) and SCREEN II (<50) as the criterion. Univariate linear regression analysis was carried out to explore associations between HGS as a key variable and undernutrition indicators. Statistically significant independent variables were included in the multivariate linear regression model. *p* value less than 0.05 was considered to be significant.

## 3. Results

The study included 300 (67%) community-dwellers and 146 nursing home residents (33%), with the mean age of 75.96 ± 7.41 years (age range from 66 to 99). Most of the participants were married (47%); had primary education (67%), specific hobbies (56%), and pensions (87%); and lived in their own homes (67%).

Clinical characteristics of the study sample are presented in Table 1. According to the classification of nutritional status by MNA, 42% of community-dwelling men and 39% of community-dwelling women were at risk for undernutrition. The risk for undernutrition was significantly higher among nursing home residing men (89%) and women (78%) (*p* < 0.001).

When nutritional status was assessed by SCREEN II, 86% of community-dwelling men and 80% of women were identified as having a high risk for undernutrition. All nursing home residents were at high risk for undernutrition (100%). SCREEN II score was significantly correlated with the MNA score (*r* = 0.684, *p* < 0.001).

An average BMI was lower in nursing home residents (26.07 ± 4.91 kg/m^2^ in men and 25.47 ± 4.93 kg/m^2^ in women) in comparison with community-dwellers (26.92 ± 3.85 kg/m^2^ in men and 28.84 ± 5.25 kg/m^2^ in women). Nursing home residents also had significantly lower other anthropometric measurements compared to community-dwelling study participants (*p* < 0.001). Majority of participants had memory difficulties (51% of community-dwelling men and 57% of women, 81% of men and 64% of women in nursing homes) (*p* < 0.001). The risk for falls per Timed Up and Go Test was lower in the group of community-dwellers (12.67 ± 7.77 sec in men, 12.89 ± 7.72 sec in women) than in nursing home residents (28.15 ± 8.71 sec in men, 28.58 ± 7.68 sec in women). Independence level in performing basic ADL was higher in community-dwellers (5.99 ± 0.08 in men, 5.99 ± 0.07 in women) compared to nursing home residents (3.70 ± 1.84 in men, 3.82 ± 1.95 in women) (*p* < 0.001). Among community-dwelling elderly, average Lawton score for men was 7.98 ± 0.16 and for women was 7.95 ± 0.39.

Nursing home residents had statistically lower HGS of right hand (19.81 ± 6.23 kgF in men, 18.55 ± 7.19 kgF in women) compared to the community-dwellers (24.05 ± 8.15 kgF in men, 20.23 ± 4.95 kgF in women) (*p* < 0.001). Statistical difference between two groups was also found in the value of left HGS (*p* < 0.001). Gender and age-specific percentiles of height were generated to describe HGS values of community-dwellers and nursing home residents in Bosnia and Herzegovina (Table 2).

**Figure 1:**
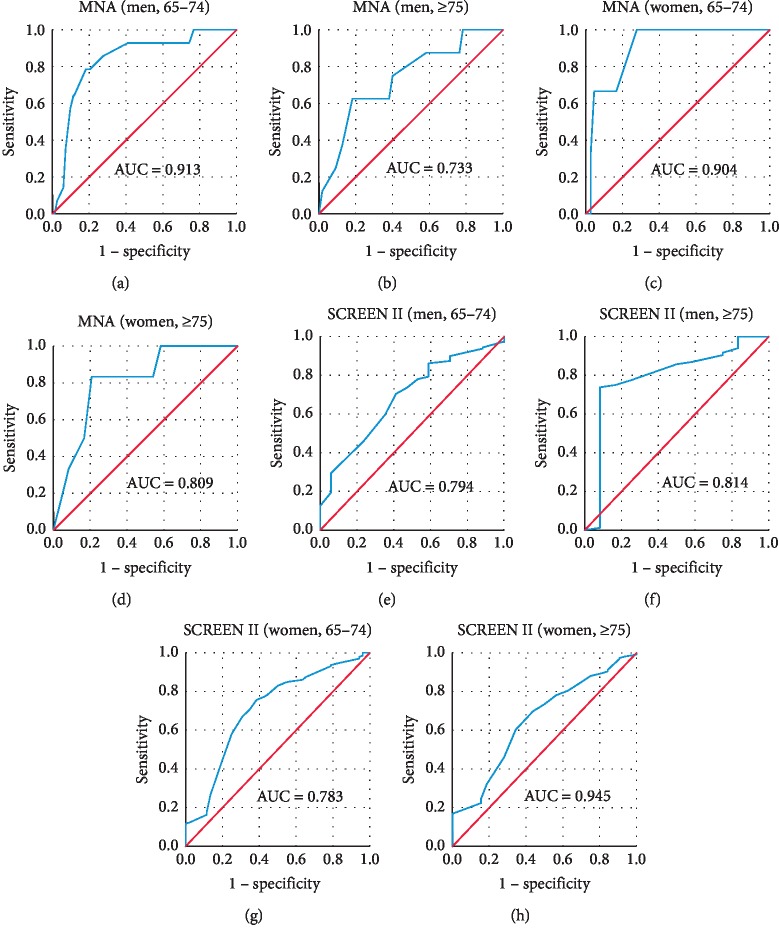
Age- and gender-specific ROC curves for identifying elderly people at risk of undernutrition according to different cut-off values for handgrip strength. AUC is indicated in figure. The AUC is >0.05 and statistically significant for all except for women older than 75 years of age.

HGS cut-off thresholds for undernutrition were 23.50 kgF (65–74 years) and 19.50 kgF (≥75 years) for men and 15.50 kgF (65–74 years) and 13.50 kgF (≥75 years) for women. For nutritional risk likelihood, cut-points were 28.50 kgF (65–74 years) and 24.50 kgF (≥75 years) for men; 24.50 kgF (65–74 years), 19.50 kgF (≥75 years for women). Except for the women older than 75 years of age in the relation to SCREEN II (AUC = 0.945, *p*=0.127), area under the curve (AUC) was >0.5 and statistically significant for all ROC curves (Table 3, Figure 1).

The results of the multivariable linear regression analysis are listed in Table 4. Female gender (*p* < 0.001), functional reach test score <15 cm (*p* < 0.001), and BMI value (*p*=0.002) were associated with low HGS in community-dwelling elderly. Individual practicing some hobbies had higher HGS values (*p*=0.015). Independent determinants on HGS value among nursing home residents were functional reach test score <15 cm (*p*=0.027), number of medical visits <3 (*p* < 0.001), and number of meals >2 (*p*=0.040).

## 4. Discussion

The current study found that low HGS is associated with undernutrition risk among the community-dwelling and nursing home-residing individuals in Bosnia and Herzegovina. The findings corroborate the results of international studies analyzing the role of HGS in nutritional status assessment [12, 13, 32]. HGS cut-off points for the undernutrition risk were described, and according to AUC values, accuracy of HGS in diagnosing undernutrition risk (stratified by age and gender) was fair to excellent.

Bosnian community-dwelling men had lower average HGS compared to their coevals in other countries, whose values ranged from 50 kgF in USA to 30.00 kgF in Portugal [33–40]. The mean value for community-dwelling women in Bosnia and Herzegovina was 20.23 kgF (dominant arm), which falls within previously published reference values; from 25.0 kgF in Sweden [33] to 16.7 kgF in Spain [41]. Several factors potentially contribute to the discrepancies in anthropometric measurements between the nationalities, including but not limited to dietary habits, physical activity level, contrasting income, perception of aging, and genetics. The percentage of individuals with a risk for undernutrition was higher among older Bosnian people than that generally found in international studies. The majority of participants in our study had primary education and consequently had low pensions. Poverty and lack of education might have influenced dietary habits and hence have led to an increased propensity to undernutrition [39].

High prevalence of risk for undernutrition among elderly in Bosnia and Herzegovina may be attributed to numerous underlying factors. The Community-based Framingham offspring cohort study described the favorable effects of high animal protein intake, but not plant protein on the preservation of HGS [42]. Increased protein intake improved overall protein synthesis and reduced protein breakdown, further leading to the restoring of protein depos and muscle strength [43]. To prevent age-related muscle loss, the ESPEN Expert group recommends a daily intake of 1.0 to 1.2 g/kg/d for sedentary elderly individuals or higher intake for active community-dwellers [44]. In Bosnia and Herzegovina, nutritional needs frequently cannot be met because many seniors face constant financial difficulties (due to low pensions), pursuing in return diets rich in carbohydrates and fat (which is much cheaper compared to healthy food) and low in vitamins and proteins. Social support networks in the country commonly struggle with limited financial and technical resources; therefore, many older adults are deprived of help and live in social isolation [45–47].

Recently published meta-analysis found that cut-off points for HGS were higher in developed regions (North America, Europe, Australia, Japan) compared to developing regions (South America, Africa and Asia) [14, 48–50]. Although variations between the countries may be attributed to the differences in physical constitution, it is not well understood how the lack of standardization in measurement protocols and statistical analyses might have affected normative data for muscle strength. Heterogeneity between countries disables identifying internationally applicable thresholds for HGS, so if this clinical marker of undernutrition is used in the assessment of nutritional status, its values must be analyzed within region-specific cut-off points [14].

All participants in our study were right-handed. In accordance with other research, dominant hands were significantly stronger compared to nondominant hands due to more frequent use in the performance of tasks and heavier workloads [51].

According to MNA, nursing home residents were at higher risk of undernutrition, while the nutritional status assessment by SCREEN II showed that both groups were at high risk for being undernourished. The correlation between the scores was high, statistically significant, and positive. Notwithstanding, validation studies showed good (MNA) and satisfactory (SCREEN II) reliability and validity of these instruments [5, 30, 52], their internal consistency in the Bosnian language was fair (SCREEN II) to good (MNA), potentially influencing the results. The magnitude of difference between the two assessments may also be related to the approach in risk assessment. MNA focused on identifying elderly individuals who are undernourished or at risk and encompassed current indicators of undernutrition [49]. SCREEN II differentiated the severity level of nutritional risk, exploring contributing factors for undernutrition development [30]. Despite MNA being the gold standard for nutritional status assessment, whether its routine administration solely improves the detection and prevention of undernutrition among older primary care patients or could detect changes in nutritional status, it is insufficiently explored. As showed in the study of Kizilarslanoglu et al., HGS may be considered as an alternative to anthropometric measurements within MNA assessment [9]. Future prospective analyses are required to determine if HGS application would improve the sensitivity of MNA through earlier detection of undernutrition.

As frailty leads to numerous adverse outcomes, measuring HGS in the context of comprehensive geriatric assessment might improve early detection of sarcopenia and the resultant time-effective introduction of therapeutic interventions to prevent disability [53]. In the year preceding research, the association between the number of hospitalizations and HGS was identified among female, but not among male, study participants. International study results vary based on design and population studies, so it is uncertain which are more decisive for the relationship between self-reported hospitalization and HGS [54–56].

This is the first study describing HGS values among older adults in Bosnia and Herzegovina. The sample represents the average Bosnian elderly population, including community-dwelling individuals, as well as institutionalized persons. Different variables were collected from participants simultaneously and consistently by the same trained rater. Undernutrition risk, previously undiagnosed, was identified as high, indicating the need for improvement in the care of elderly populations in primary health care. In regards to practice implications, the study highlighted the utility of measuring muscle strength across the elderly population. Although HGS cannot be used as a solemn indicator of undernutrition, it could provide valuable information on undernutrition risk. The HGS cut-off points for undernutrition risk should be considered within age, gender, and height. Measuring HGS may help family physicians or other primary care workers (frequently only providers included in the care for older adults) identify older adults who would benefit from more comprehensive nutritional assessment and nutritional therapy programs. The results also propose that it is necessary to improve the quality of care for nursing home residents, who are often marginalized in the healthcare system of Bosnia and Herzegovina [57]. To improve the health of the elderly patients and change their attitude towards nutrition, primary care providers should pay more attention in their daily practice to counseling of older individuals about a healthy, balanced diet.

However, several limitations need to be considered. This study design was cross-sectional, exploring the association between variables, but not the cause-effect or direction of the relationship. Although we measured the HGS using the recommended protocols, the method might be different compared to the methods other researchers used, what might have influenced the results. Using HGS as a marker of undernutrition risk is difficult in cognitively impaired people and individuals with severe musculoskeletal disorders. High prevalence of high undernutrition risk might have had impact on cut-off values for HGS.

There may also be other variables influencing HGS or undernutrition status that were not explored in the current study. Further studies are required to analyze and validate if internationally acceptable cut-off points of HGS could be used as a diagnostic criterium for undernutrition risk in clinical practice. It should also be investigated whether HGS could replace robust assessment tools as an undernutrition screening instrument. To minimize variation in HGS measuring and facilitate comparison of international data, research exploring standardized measurement protocol needs to be explored in the future.

## 5. Conclusion

According to MNA and SCREEN II, elderly individuals in Bosnia and Herzegovina are at high risk of undernutrition. HGS could be the useful clinical indicator of nutritional status within the routine comprehensive geriatric assessment. A region-specific definition of cut-off values, stratified by age and gender, is necessary to identify the population at undernutrition risk. The results of the current study contribute to data on HGS values of older Bosnian adults; however, further validation studies need to be conducted.

## Figures and Tables

**Table 1:** Sociodemographic and clinical characteristics of study participants.

Variable	Community-dwellers		Nursing home residents		*p* ^*∗*^
	Men (*n* = 141)	Women (*n* = 159)	Men (*n* = 54)	Women (*n* = 92)	
Age, years (SD)	73.78 (6.74)	72.69 (5.60)	81.30 (6.93)	81.80 (6.18)	**<0.001**
Education, *n* (%)					
Primary	102 (53)	198 (79)	40 (74)	75 (82)	**<0.001**
High school	73 (37)	50 (20)	11 (20)	16 (17)	
University	20 (10)	3 (1)	3 (6)	1 (1)	
Chronic diseases, *n* (SD)	2.30 (1.71)	2.20 (1.70)	2.93 (1.16)	3.17 (1.03)	**<0.001**
Medical visits, *n* (SD)	9.38 (3.98)	9.58 (4.47)	4.89 (2.56)	5.03 (2.43)	**<0.001**
Hospitalizations, *n* (SD)	0.46 (1.13)	0.40 (0.77)	0.43 (0.81)	0.43 (0.70)	**0.034**
Medications, *n* (SD)	3.82 (2.23)	3.94 (2.28)	6.61 (2.46)	7.32 (2.16)	**<0.001**
Body mass index (kg/m^2^)	26.92 (3.85)	28.84 (5.25)	26.07 (4.91)	25.47 (4.93)	**<0.001**
Weight (kgF)	82.15 (13.08)	75.08 (13.97)	73.35 (15.00)	64.91 (13,78)	**<0.001**
Height (cm)	174.61 (7.35)	161.55 (7.13)	167.70 (9.31)	159.5 (8.01)	**<0.001**
Waist circumference (cm)	100.95 (12.10)	94.96 (12.93)	95.38 (14.66)	90.18 (11.24)	**<0.001**
Skinfold measurement (mm)	14.86 (7.27)	18.64 (6.72)	15.79 (2.64)	14.93 (2.85)	**<0.001**
Calf circumference (cm)	32.53 (4.13)	32.66 (6.29)	29.52 (3.12)	28.12 (2.89)	**<0.001**
Mid-arm muscle circumference (cm)	27.60 (3.50)	27.49 (3.67)	25.29 (2.65)	25.44 (3.33)	**<0.001**
SCREEN II					
High risk of undernutrition	121 (86)	127 (80)	54 (100)	92 (100)	**<0.001**
Moderate risk of undernutrition	18 (13)	26 (16)	0	0	
Without risk	2 (1)	6 (4)	0	0	
MNA					
Without undernutrition	82 (58)	96 (60)	2 (4)	7 (8)	**<0.001**
With risk of undernutrition	59 (42)	62 (39)	48 (89)	72 (78)	
Undernutrition	0 (0)	1(1)	4 (7)	13 (14)	
6-CIT test					
No cognitive Impairments	96 (49)	109 (43)	10 (19)	32 (26)	**<0.001**
Cognitive impairments	99 (51)	142 (57)	44 (81)	60 (64)	
Timed up and go test (sec)	12.67 (7.77)	12.89 (7.72)	28.15 (8.71)	28.58 (7.68)	**<0.001**
Functional reach test (cm)	31.73 (11.97)	29.62 (10.47)	16.69 (6.66)	15.91 (6.01)	**<0.001**
Katz index, score (SD)	5.99 (0.08)	5.99 (0.07)	3.70 (1.84)	3.82 (1.95)	**<0.001**
HGS-left arm (kgF)	21.38 (6.61)	17.54 (4.82)	15.44 (6.20)	14.41 (6.70)	**<0.001**
HGS-right arm (kgF)	24.05 (8.15)	20.23 (4.95)	19.81 (6.23)	18.55 (7.19)	**<0.001**
Albumin (g/l)	45.80 (2.36)	46.00 (7.17)	44.23 (4.89)	43.65 (4.35)	**0.047**
Ferritin (ng/mmol)	192.19 (142.54)	99.58 (70.22)	212.47 (164.99)	149.14 (114.27)	**0.028**
Total cholesterol (mmol/l)	5.34 (1.06)	5.55 (1.07)	5.41 (0.96)	5.10 (1.22)	0.236
Hemoglobin (g/l)	145.73 (13.58)	131.76 (12.08)	131.55 (19.61)	120.21 (15.31)	**<0.001**

Data for continuous variables are presented as mean ± SD. Categorical variables are presented as numbers and percentages. Screen II-seniors in the community: risk evaluation for eating and nutrition, version II among octogenarians. MNA-SF, Mini Nutritional Assessment-Short Form. 6-CIT, Six-Item Cognitive Impairment Test HGS-handgrip strength. ^*∗*^Significant *p* values are in bold.

**Table 2:** Handgrip strength values of Bosnian elderly population by age, gender and height.

Handgrip strength (kgF)													
Age. years	Height. cm	*N* (%)	Mean (SD)	85% of mean	Min	Max	Percentile 10	Percentile 15	Percentile 25	Median	Percentile 75	Percentile 85	Percentile 90
Men, *n* = 195													
65–74	<161.0	5 (5.2)	21.8 (4.9)	18.5	18.0	30.0	18.0	18.0	18.0	21.0	22.0	30.0	30.0
	161.0–167.0	7 (7.3)	24.2 (8.6)	20.5	14.0	48.0	17.0	17.0	18.0	19.0	30.0	35.0	40.0
	≥167.0	84 (87.5)	29.4 (10.9)	25.0	14.0	45.0	14.0	19.0	19.0	30.0	38.0	38.0	45.0

75–84	<161.0	12 (18.2)	20.2 (6.0)	17.1	13.0	35.0	15.0	15.0	16.5	19.0	21.0	28.0	28.0
	161.0–167.0	7 (10.6)	22.1 (6.2)	18.8	11.0	29.0	11.0	19.0	19.0	22.0	27.0	27.0	29.0
	≥167.0	47 (71.2)	23.0 (7.4)	19.6	10.0	43.0	17.0	18.0	18.0	20.0	29.0	30.0	35.0

≥85	<161.0	3 (9.1)	17.3 (6.8)	14.7	12.0	25.0	12.0	12.0	12.0	15.0	25.0	25.0	25.0
	161.0–167.0	5 (15.2)	18.0 (2.1)	15.3	15.0	21.0	15.0	15.0	18.0	18.0	18.0	21.0	21.0
	≥167.0	25 (75.8)	19.8 (6.2)	16.9	10.0	32.0	10.0	12.0	18.0	19.0	22.0	28.0	30.0

Women, *n* = 251													
65–74	148.0–153.0	4 (3.6)	19.3 (5.9)	16.4	15.0	28.0	15.0	15.0	16.0	17.0	22.5	28.0	28.0
	≥153.0	107 (96.4)	21.1 (5.4)	18.0	7.0	35.0	15.0	17.0	18.0	20.0	25.0	26.0	30.0

75–84	<148.0	7 (6.4)	16.6 (5.9)	14.1	10.0	35.0	10.0	10.0	10.0	18.0	20.0	20.0	26.0
	148.0–153.0	12 (10.9)	18.0 (4.7)	15.3	12.0	26.0	13.0	13.0	15.0	17.5	20.0	20.0	20.0
	≥153.0	91 (82.7)	19.4 (6.0)	16.4	6.0	30.0	12.0	15.0	17.0	19.0	22.0	25.0	29.0

≥85	<148.0	1 (3.3)	10.0 (6.3)	8.5	10.0	10.0	10.0	10.0	10.0	10.0	10.0	10.0	10.0
	148.0–153.0	3 (10.9)	13.7 (3.5)	11.6	10.0	17.0	10.0	10.0	10.0	14.0	17.0	17.0	17.0
	≥153.0	26 (82.7)	16.7 (6.9)	14.2	8.0	35.0	11.0	11.0	12.0	15.5	19.0	25.0	25.0

**Table 3:** Cut-points of HGS values to detect undernutrition risk on the basis of MNA and SCREEN II.

Variable		Age	AUC	SE	*p*	95% CI	Cut-point (kgF)	Sensitivity (%)	Specificity
Men	MNA	65–74	0.913	0.060	**0.014**	0.796–1000	23.50	100	65.7
		≥75	0.733	0.094	**0.034**	0.548–0.918	19.50	87.5	67.3
	SCREEN II	65–74	0.794	0.077	**0.001**	0.642–0.948	28.50	85.7	50.0
		≥75	0.814	0.071	**0.007**	0.674–0.953	24.50	83.1	42.9

Women	MNA	65–74	0.904	0.057	**0.017**	0.792–1000	15.50	66.7	50.0
		≥75	0.809	0.092	**0.021**	0.630–0.988	13.50	83.0	48.6
	SCREEN II	65–74	0.783	0.075	**0.033**	0.635–0.931	24.50	77.8	52.4
		75–84	0.945	0.024	0.127	0.897–0.992	19.50	65.0	50.0

MNA-SF, Mini Nutritional Assessment-Short Form. Screen II, seniors in the community: risk evaluation for eating and nutrition, version II among octogenarians. Significant *p* values are in bold.

**Table 4:** Multivariate linear regression analyses for HGS.

Variable	HGS			
	*B*	Std. error	*t*	*p* ^*∗*^
*Community-dwellers*				
Female gender	−3.260	0.70	−4.654	<0.001
Functional reach test	0.270	0.032	8.412	<0.001
BMI	0.074	0.024	3.064	0.002
Having hobby	1.690	0.691	2.445	0.015

*Nursing home residents*				
Functional reach test	0.186	0.083	2.238	0.027
Number of medical visits, <3	−0.856	0.215	−3.987	<0.001
Number of meals, >2	1.366	0.658	2.076	0.040

BMI, body mass index. HGS, handgrip strength. ^*∗*^Only statistically significant variables are presented.

## Data Availability

The research data used to support the findings of this study were supplied by Darko Marinkovic under license and so cannot be made freely available. Requests for access to these data should be made to Dr. Jelena Pavlovic, Studentska 5, 73000 Foca, Bosnia and Herzegovina, pjelena551@gmail.com.
